# Compressed Air in Dentistry

**Published:** 1904-11-15

**Authors:** George Zederbaum

**Affiliations:** Charlotte, Mich.


### DENTAL REGISTER.

Vol. LVIII. November 15, 1904. No. 11.

### COMPRESSED AIR IN DENTISTRY.

BY GEORGE ZEDERBAUM, D.D.S., CHARLOTTE, MICH.

Read before the Michigan Dental Association, June 28, 1904.

It is my purpose to bring before you the many advantageous uses of compressed air in the practice of dentistry.

We shall first consider the apparatus necessary to obtain and maintain a certain pressure of compressed air. In my opinion, the best apparatus is the one described and illustrated by Dr. H. C. Register, of Philadelphia, in the April number of the *Dental Brief*, and on exhibition here with Sibley's goods. It consists of a small pump which operates by being connected by a pulley to an electric motor. When the motor is set in motion, the pump compresses the atmospheric air into a storage tank which, by means of an air gauge, accurately registers the pressure of air stored. From the storage tank leads the outlet pipe guarded by a suitable valve. Unfortunately the use of this outfit is limited to those who are located where a street electric current is obtainable. The other apparatus, and the one we all can have, is the one depending upon the water-supply, and this is available in almost all towns. No very high pressure is necessary. Here we have an "air pump which is attached to the city water-supply pipe, and which keeps a desired pressure of air in the storage tank automatically." A very small pump, such I as just described, will maintain from thirty to forty pounds pressure of air for a very long period. The manufacturers of these pumps claim that only one pound of pressure is lost by friction, that is, your compressed-air tank when kept full, will register just one pound less than your water supply registers. The temperature of air so obtained is about twenty-five degrees Fahrenheit higherr than that of the water at the same time. Either apparatus described may be had for some twenty-five of thirty dollars. They occupy but very little little room, and in case of water-motor and tank, these can be stored in the cellar and air piped up to the most convenient point.

There are many other apparatuses on the market, some with hand and others with foot pumps, but I would have the same objections to these that I would have to a common rubber bulb atomizer, namely, insufficient capacity and insufficient pressure of air for prolonged use. So much for the apparatus.

That the compressed air is becoming more and more a necessity for us and not merely a luxury, as many may suggest, one has to look into some of our valuable dental publications. There are quite a few of the thinking practitioners considering the installation of compressed-air outfits in their offices. It is only the other day that I read an advertisement in one of the dental journals of a modern dental office for sale; everything complete, electric engine, lathe, compressed-air outfit, etc. This is my sentiment exactly. No office is complete and up-to-date in every sense of the word, without its compressed-air supply. Now let us consider the many varied uses for this air. I will suggest only those that I have had experience with during the last two years. There may be many more advantageous uses for it, but I leave them for others to demonstrate. All I wish is to show its value to us, whether it be for the laboratory or for the operating room or for the extracting room.

Preparing and filling of sensitive cavities with plastic filling materials becomes an easy task when the cavity is thoroughly dry; a stream of compressed air will dry the cavity and will enable the operator to do better and quicker work. Let us take a large buccal cavity in a lower molar, involving say middle and gingival thirds occluso-gingivally and the middle-third mesio-distally. These, we will all agree, are by no means easy to fill as they should be filled, especially so in cases where one has to do it without the use of rubber dam. Such submarine fillings are never satisfactory. A current of air applied for the period of two minutes will, in most mouths, entirely dry the seat of operation, and after the filling is inserted, it will require some effort on the part of the patient to bring about the normal moisture.

In preparing any and all cavities, we are obliged to stop frequently and blow out the chips obscuring the point of operation, and not infrequently do our patients complain of heat generated by a rapidly-revolving bur. Let us take a very small rubber tube, the diameter of the opening of which need not be over one thirty-second of an inch, and wire it around the engine cable down to the point of the handpiece; now turn on your air pressure and go ahead with the drilling. The current of the air will not only keep the point of operation in constant sight and free from chips, but will also prevent the bur from heating and thereby will, necessarily, lessen pain and duration of operation. Nowadays we are after modes and means calculated toward lessening degree and amount of pain, and toward doing work with expediency, and for this purpose alone the compressed air is a desirable feature. Should it be desired to heat the air before it reaches the cavity, that is also easily accomplished, for all that is necessary is for us to state our needs and wants and there are many ingenious minds among us to devise such instruments and accessories as may be needed and are not yet in the market.

In taking impressions and bites in wax or in gutta-percha, or in various modeling compounds requiring heat for their plasticity, all one has to do after their introduction into the mouth is to turn on the compressed air and cool off the mass in a minute. You will have a sharper impression or bite as the result; patient will not get tired out and spoil the impression by involuntary closing the mouth or changing the relative position of the jaws in case of bite. Again you are saving time, and the trouble of preparing another "hot cake" is obviated.

In treatments of empyema of the maxillary sinuses, or of a wound after a root incision, or of any other injury about the mouth, where the parts to be treated are remote from sight and easy reach of the operator, and where a thorough medication is required, whether it be of a liquid or of a powder nature, here again the best mode of application is easily solved. If it be a liquid, a pointed atomizer point will, by means of gentle but steady pressure of air, do the work more effectively than when applied by old methods. If it be some powder medicament, I would use a glass pipette into which I gather a few pinches of this powder; the free end of this pipette is attached to the air valve and the opposite end is inserted just at the opening of the wound. Then, in the language of kodakers, "you touch the button, and it does the rest." The powder so applied will be distributed all over the wound more uniformly, the deepest portions being reached, will quickly stimulate healthy granulations. These deepest portions, as a rule, are slighted owing to inaccessibility of the parts; but here the force can be exerted as the case may require; there is no danger of the powder so distributed coming back into the operator's face as in the case of a clown in Barnum's circus, who, upon being instructed to give some pulverized medicament to a sick donkey, made a large cardboard pipe and filled it with the prescribed powder. Then upon inserting one end of the pipe into the donkey's mouth and the other end into his own, he took a long breath preparatory to blow the powder into the donkey's mouth, but the donkey's olfactory nerve became tickled and he blew first! It is needless to say that the attempted operation was not a success. Had the clown use the compressed-air outfit all would have gone well.

In treating pyorrhea in all its varieties and stages and in many diseases of the pericementum, one saves much time and effects a permanent cure considerably quicker by the use of a compressed-air atomizer and treating the diseased parts directly by daily spraying with proper medicaments. Marshall, in his work on "Operative Dentistry," suggests the use of atomizers for this purpose. Here again the compressed-air apparatus enables one to send uninterrupted streams as strong or as weak as the case in hand may demand into the deep tissues, down into pus pockets where it is most needed.

To deodorize malodorous mouths before commencing an operation is a desirable feature beyond dispute; here again comes the valuable and efficient use of compressed-air handy.

While touching upon deodorization, I wish to remark that not infrequently our operating rooms have peculiar "dental odors" about them. Iodoform, carbolic acid, creosote, oils of cassia, cloves and other essential oils and highly-volatile substances, all add to the office, in the mind of many sensitive patients, still more repugnancy than they already have from fear. It is my habit to spray the room with a weak solution of spirits of lavender; fumigate, so to speak, the entire breadth and length of the operating room. Compressed air does this admirably and the room smells sweet and fresh and the air is by far more agreeable.

How many bridges and crowns have to be reset in our practice, and all because in the majority of cases, they were put on while saliva was running freely in and about the abutments? With a steady compressed-air current I can dry up the mouth so that a bridge or a crown can be set under absolutely-dry conditions, thereby assuring their stability.

Have you a long soldering job? Here also take your compressed-air hose and attach to it your blow-pipe, and the absolute ease with which the soldering can be done is surprising. Furthermore, no checking of porcelain is apt to occur when the heat, as in this case, is steadily applied.

After giving nitrous oxide or any other general anesthetic, patient is brought to quickly by application of a strong force of compressed-air directed squarely toward his face.

Very often patients faint at extraction; compressed air, applied as above, revives them at once.

The use of local anesthetics by means of injections with the hypodermic needle is objectionable to many patients; still they will not object if you "freeze the gums" by the local abstraction of heat, directing finely-divided streams of ether or rhigolene or other such remedy by means of the compressed-air apparatus. Here also it is by far more effective than atomizers operated by hand or foot bellows. The stream is steady, and consequently less of the drug is used and the required numbness is obtained in much less time.

After extracting, I often check the hemorrhage by spraying into the alveoli some suitable styptic; here again I used the compressed-air outfit.

The problem of interesting the children, while doing some painful operation for them, is a hard one; I often let the child help me by holding the compressed-air valve and keeping the cavity blown out. They like the idea of helping you, their mind is away from the seat of trouble and you accomplish better and quicker work with but an occasional involuntary flinch from them.

No doubt there are many and many more useful ways of utilizing the compressed air, once it be installed in our offices. They become apparent as one uses the apparatus and becomes acquainted with it. When I first commenced to use it, I applied it only where I wanted to prepare a difficult cavity without the use of the rubber dam. Since then, all the other various uses I have mentioned followed naturally, each in its respective turn. I know that but very few have ever used compressed air; many have never thought of it. I wish all would try it and become convinced and, as I have said before, add more to the already numerous enough list of advantageous uses of compressed air in dentistry to commend its intrinsic value.

### DISCUSSION.

DR. C. P. WOOD: I have made much use of compressed air, and can cordially endorse all that the essayist has said in relation to its valuable and varied uses. Especially is it valuable in drying the gums for setting crowns and bridges. For this purpose I use an alcoholic spray, as this produces a more thorough drying and stops the oozing of blood or serum. I remember once using it to keep out the saliva from a small puncture of dam until I had completed the filling. It is also valuable in disinfecting the mouth or preparing it for operative treatment or medication. The essayist may well be enthusiastic about it. Like many other good things, it will not do everything, and it may work injury sometimes by using it with excessive pressure or prolonging the application unduly. It can be made one of the handiest of our office equipments if used with judgment.

DR. LEGRO: I have used compressed air considerably during the past four years, and should not like to be without it in my office, as it is so generally useful. You will find new uses for it every day. It all depends on whether one has an effective apparatus, whether he really gets all his benefits or not. I use and like the water-motor pump as it does not require so much attention as the electric pump. I have had no trouble or expense from my motor since putting it in, four years ago. It is important that the plant be properly installed. A good water force is essential, and a large tank, such as a forty-gallon hot-water boiler, which is used in connection with the kitchen range. This should be absolutely tight. It can be made so by pouring a gallon of asphaltum varnish into it and rolling the cylinder about until the whole interior is covered with the varnish; the excess can then be drained out, and, when the varnish has hardened, it can be used. A proper valve should be added to control the pressure and well-fitted and connected pipings for conducting it to the chair or bench where it is to be used. I don't find it desirable to apply cold air to sensitive dentine. I have much better success by using an electric heater in connection with the blast, as this does not irritate the tooth, but has rather a desensitizing function.

DR. ZEDERBAUM: I wish to have you note the fact mentioned in the paper that the compressed air is from twenty-eight degrees to thirty degrees warmer than the water which pumps it. If the tank is placed in a warm room the air will, of course, have practically room temperature. Except that if sprayed into the cavity it produces some lowering of temperature, because of the evaporation of moisture from the tooth and the current which agitates the air in the neighborhood of the tooth. I trust all who can will give it a good trial, as I am confident any one beginning its use will continue it.

